# Role of Occupation in Shaping Cancer Disparities

**DOI:** 10.3390/cancers14174259

**Published:** 2022-08-31

**Authors:** Giulia Collatuzzo, Federica Teglia, Paolo Boffetta

**Affiliations:** 1 Department of Medical and Surgical Sciences, University of Bologna, 40138 Bologna, Italy; 2 Stony Brook Cancer Center, Stony Brook University, Stony Brook, NY 11794, USA

**Keywords:** cancer disparities, occupation, workers, education, ethnicity, cancer screening, lung cancer, occupational exposure, mediation analysis

## Abstract

**Simple Summary:**

The investigation of cancer disparities is of major importance. In this paper, we address this issue through the occupational point of view, trying to capture how work and its related factors impact on cancer inequalities. The data we provide may increase awareness relevant to cancer control, and stimulate further studies aimed at the identification of the occupational determinants of cancer disparities and the quantification of their role.

**Abstract:**

Cancer occurrence is characterized globally by profound socioeconomic differences. Occupation is a fundamental component of socioeconomic status. In this review, we discuss the role of occupation as a determinant of cancer disparities. First, we address the issue of participation in cancer screening programs based on income, health insurance, occupational status and job title. Second, we review the role of occupation in contributing to disparities by acting as a mediator between cancer and (i) education and (ii) race/ethnicity. Lastly, we analyze data from a multicenter case−control study of lung cancer to calculate the mediating role of occupational exposure to diesel exhaust, silica and welding fumes in the association between education and lung cancer. By addressing the complex paths from occupation to cancer inequalities from multiple points of view, we provide evidence that occupational-related characteristics, such as income, health insurance, unemployment and hazardous exposures impinge on cancer control and outcomes. The increasing awareness of these aspects is fundamental and should lead to public health interventions to avoid inequalities rising from occupational factors.

## 1. Introduction

Cancer incidence and survival are affected by sociodemographic inequalities related to factors such as gender, ethnicity, education, place of residence and occupation. Working position can indeed influence cancer occurrence, both in direct (exposure to occupational carcinogens) and indirect ways (through income, health education, access to screening programs, working hours and workload, free time and stress level). Temporal trends of cancer rates appear to be dynamic, reflecting the evolution and progressive changes of society and the environment. The introduction and regulation of exposure to carcinogens at the workplace, the promotion of personal protective equipment (PPE) use and the continuous update of surveillance protocols have followed the improvement of knowledge regarding occupational cancer hazards. This has been especially effective at downscaling the incidence of cancer among workers highly exposed to carcinogens, such as construction, metal and wood workers, miners, painters and other groups employed in manual jobs. 

The less affluent sector of the population has historically been exposed to high levels of carcinogens [1], such as chimney sweeps being exposed to soot, later causing scrotal cancer [2], oil shale workers being highly affected by skin cancer [1,3] and dyers being exposed to aromatic amines associated with bladder cancer [4]. Asbestos’ experience gives the most powerful example of the benefit gained through the efforts of occupational medicine, which reduced the burden of cancer in subsequent generations of workers [5]. Other occupational carcinogens have also been controlled. For example, in France, a 17.5% decrease in the prevalence of carcinogenic, mutagenic and reprotoxic chemical exposure has been observed in the period from 2003–2010, also resulting in a lower frequency and intensity of exposure [6]. However, this reduction was not homogeneous between employee categories, with a lesser effect observed for blue collar and service workers, those working in smaller companies and precarious subgroups, such as apprentices.

Cancer disparities are a major health issue and may be more impactful in certain occupational categories. This review aims to address the role of selected occupation-related factors in shaping cancer disparities in three domains: screening uptake among workers, the role of sociodemographic variables other than occupation and occupational exposure to carcinogens. First, we focused on the adherence to the best-established cancer screening programs, namely colorectal, cervical and breast cancer screening. Next, we reviewed the evidence of a mediating role of race/ethnicity and educational level on cancer risk due to occupational exposure. Lastly, we conducted an original analysis on occupational exposure to diesel exhaust, welding fumes and silica dust as mediators in the association between education and the risk of lung cancer based on a multicenter study [7].

## 2. Cancer Screening by Income, Health Insurance and Occupation

Educational level, occupational position and income are correlated in defining the socioeconomic status (SES) of the individual [8]. As a single component cannot fully capture the complex features of SES, many epidemiologic studies consider a combination of these aspects when measuring their impact on cancer risk. Despite some inconsistencies between educational level and income, in general, subjects with a high literacy level held a high proportion of managerial, high-professional jobs, which entail higher wages [9]. Income, which largely reflects the hierarchy of working categories and positions, is a major factor determining health disparities. Health insurance is the single factor most effecting access to health services and screening programs, resulting in a disadvantage for minorities defined by race, ethnicity, education or employment [10]. Indeed, for Korean people of lower income, the use of cancer screening is reduced by about 20% (80% vs. 60% based on household income), limiting the possibility of an early diagnosis of cancer [11]. Table 1 [11,12,13,14,15,16,17,18,19,20] reports data from selected studies to illustrate how these disparity-related factors affect participation in cancer screening in different countries.

As mentioned, studies are consistent in showing a direct relation between the level of education and income and cancer screening participation. These discrepancies may arise not only from differences in wealth and, consequently, access to health services, but also from differences in knowledge, attitude and behavior about cancer risk and prevention. For example, holding a job was independently associated to better knowledge of (OR 2.37, 95% CI 1.12–5.04) and willingness to perform (OR 2.32, 95% CI 1.09–4.75) cervical screening compared to being a housewife in Pondicherry, India [21]. 

A comparable difference can be found when considering occupational position, as the type of job reflects income and in large part, educational level. Moser et al. described a lower rate of participation in breast cancer screening in Great Britain based on welfare indicators, but occupation was found not to be associated to it [22]. Also, low income was not reported to be related to low adherence to cervical cancer screening [22]. Broberg and coauthors identified a low income among the factors associated with low participation in cervical cancer screening, together with low education and unemployment [13]. 

These discrepancies are observed both in more developed and less developed countries. For example, a cross-sectional study conducted in 2015 in Ethiopia found 54.7% of women with higher income to be aware of cervical cancer screening, compared to 33.3% of those with lower income [23]. Conversely, a study conducted in Zimbabwe found no relationship between occupation and income and cervical cancer screening use [19]. 

Beyond working-related differences, employment status itself deeply affects access to healthcare, including cancer screening. Homeless adults are a subgroup of the population with a high prevalence of unemployment and a lack of insurance, combined with a high exposure to environmental carcinogens [24]. In a study from Boston, homeless individuals experienced an increased cancer mortality and were diagnosed at a more advanced stage compared to the population at large, reflecting a lower rate of participation to cancer screening programs [25]. Indeed, homeless individuals were reported to be less likely to use cancer screening services in several studies [26,27,28,29]. Precarious employment was also associated with lower participation in cancer screening [14].

Overall, the evidence supports the hypothesis that occupational-related factors such as working status, job placement, salary and health insurance impact participation in cancer screening programs. While on the one hand economic intervention may be needed, including compensation for subjects undergoing screening [30], on the other hand, the workplace appears to be an ideal setting to promote and implement cancer screening protocols [31]. This could help enhance the involvement of the population in cancer screening by targeting people of a working age and possibly their families. In this regard, in order to make such an intervention cost-effective, it would be important to select high-risk workers [32], such as those exposed to specific carcinogens [33] or with individual risk factors (e.g., family history of cancer) [34,35,36]. Programs for female cancer screening would also have a large impact if they were offered at the workplace [37,38], thus facilitating the contact with health services and enabling women to better adhere to breast and cervical cancer screening, which start early in life. 

Additionally, it might be more efficient to implement health education interventions in the workplace rather than in the community [37,39,40]. 

Despite this evidence, to date, occupation-based cancer screening is not common. With the implementation of comprehensive occupational health programs, exemplified by the Total Worker Health Program recently released by the National Institute for Occupational Safety and Health (NIOSH) [41], which includes among its aims, “comprehensive screenings for work-related and non–work-related health risks”, occupation-based cancer screening will probably play a growing role in the health care system of many countries. If, on the one hand, this additional intervention will help enhance the users of cancer screening, also directly involving occupational groups which are less commonly adherent to health check-ups; on the other hand, the occupation-based approach will leave out the unemployed, leading to an increase in disparities by occupational status. A potential way to limit this problem would be the extension of the screening program to the household, possibly enabling contact with unemployed members of the workers’ households.

## 3. The Mediating Role of Occupation in the Association between Race/Ethnicity and Education and Cancer Risk

The implication of socioeconomic factors for cancer incidence has long been discussed. While their association is corroborated by numerous studies, it is not supported by biological and physio-pathological mechanisms. Rather, the strong relationship between SES and cancer is due to additional factors which correlate with socioeconomic level. SES is commonly conceived as reflecting the combined effect of education, occupation and income. These can also correlate with additional environmental and lifestyle risk factors, such as tobacco smoking, alcohol drinking, poor diet and air pollution. However, the causal direction of this relationship, that is, whether lifestyle factors are effects or determinants of SES, may not be easy to distinguish. Occupational disparities were reported to be more evident among cancers related to smoking, alcohol and reproductive factors [42]. 

An approach to quantify the proportion of the association between SES and cancer risk, which is due to other factors, is mediation analysis. Here, we review the potential role of occupation in mediating the association between two important contributors of social inequalities to cancer risk, race/ethnicity and education (Table 2 [43,44,45,46,47,48,49,50]). 

### 3.1. Racial and Ethnic Minorities

The effect of race-related job discrimination in determining the epidemiology of occupational-related cancer has been discussed by Michaels (1983) [43], who noticed a higher cancer mortality of Black workers compared to White co-workers in United States industries. Indeed, one factor was the higher likelihood of employment in high-risk jobs, characterized by exposure to carcinogens and other pathogens. Black workers have traditionally been assigned to more inferior jobs and tasks in terms of skill requirement, environmental characteristics, exposure and workload than White workers, with consequences in terms of morbidity and mortality. At the same time, Blacks are scarcely investigated in occupational studies and many studies do not represent a large enough group to be addressed separately from Whites. 

One possible explanation of the role of race/ethnicity as an indicator of disease disparities is the different distribution of occupational hazards across these groups. In a study of steelworkers, occupational patterns, rather than race, explained the high mortality from cancer, which was mostly concentrated in the coke oven workers, characterized by exposure to several carcinogens, one being benzo[a]pyrene [43]. The risk of lung cancer among those working in coke ovens for five or more years, 70% of whom were Black, was ten times higher than unexposed steel workers; a higher risk was also reported for kidney and skin cancer. Overall, in this cohort, 89% of Blacks worked in coke ovens compared to 31% of Whites. 

Michaels listed several additional examples of industries where the distribution of employees appeared to be inequal based on race, including the rubber industry, foundry, shipyards and agriculture [43]. 

A recent investigation of racial disparities was conducted within the National Lung Screening Trial (NLST) cohort [44], showing both higher proportions of lung cancer and exposure to carcinogens including asbestos and silica in Blacks than in Whites. The authors reported an overrepresentation of racial and ethnic minorities such as Blacks and Hispanics in material-handling jobs, reflecting a disproportionately large percentage of the workforce exposed to industrial carcinogens. 

Consistent results are available from recent studies, where ethnic minorities are reportedly more exposed to occupational carcinogens than other workers [45,46]. 

### 3.2. Educational Level

Worse cancer outcomes and higher incidences are seen among lesser educated individuals [50,51,52]. This often corresponds to less skilled, more manual and more hazardous occupational positions, such as miners, construction, agricultural and steel workers and welders, given the strong correlation between literacy and job type.

The Finnish cancer registry was used by Pokhrel and coauthors to conduct an analysis on cancer outcomes by level of education, classified into three groups (<10, 10–12, >13 years of education) [47]. Since cancer survival is related to effective treatment and overall care of the patient, the fact that social position relates to survival implies inequalities in access to care. Another explanation is that lesser educated subjects could be diagnosed at more advanced stages than more highly educated people. In addition, cancer survival will also depend on factors like tobacco smoking, alcohol use and nutritional status, which may also influence treatment options. In this study, survival was consistently higher for patients with higher education and lower for those with lower education. The differences were, in part, attributable to less favorable distributions of tumor stages in the lower education categories. In 1996–2005, 4–7% of the deaths in Finnish cancer patients could have potentially been avoided during the first 5-year period after diagnosis if all the patients had the same cancer mortality as the patients with the highest educational background. This proportion would have been as high as 8–11%, if, in addition, mortality from other causes had been the same as in the highest educational category. 

The analysis of US mortality data for 2001 from the National Center for Health Statistics (NCHS) evidenced a higher risk of all-cancer mortality in subjects with <=12 years compared to those with >12 years of education, irrespective of race and sex, with stronger disparities among men [52]. This analysis also showed that education and race were independent risk factors of all-cancer mortality [52]. 

Moreover, for both men and women, the magnitude of the mortality ratio between Black and White subjects was higher among poorly educated individuals than individuals with higher education [52].

In an analysis of lung cancer in a European cohort, occupational exposure to asbestos, heavy metals and *polycyclic aromatic hydrocarbons* (PAH) explained 14% of the socioeconomic inequalities, defined based on education, after adjustment for smoking and fruit and vegetable consumption [48]. A plausible hypothesis is that higher exposure to risk factors explains the higher incidence of lung cancer in low socioeconomic groups. In other words, the risk factors are seen as intermediate variables or mediators between education and the onset of lung cancer. In particular, the authors found that the indirect effect of smoking varied by educational level, with the strongest indirect effect observed for those with the lowest education. 

### 3.3. An Example of Mediation Analysis: Education, Occupational Carcinogens and Lung Cancer Risk

Among the cancers which show strong socioeconomic inequalities, there are those of the lung, bladder, stomach and larynx [53]. We chose to explore the association between education, occupational exposures and lung cancer because of the importance of the neoplasm and its strong association with both risk factors. This approach has been followed in some previous analyses [8].

To this aim, we analyzed data from a multicenter case−control study of lung cancer conducted in six Central and Eastern European countries, including Czech Republic (three centers), Hungary (five centers), Poland (two centers), Romania (one center), Russia (one center) and Slovakia (three centers) [7].

Incident cases of lung cancer were enrolled from cancer centers and specialized hospitals, controls were hospital patients admitted for conditions unrelated to environmental or occupational factors or tobacco smoking. Cases for this study were patients aged 20 to 79 years newly diagnosed with cytologically or histologically confirmed lung cancers from 1998 to 2001. In all centers but Warsaw (Poland), controls were recruited from the same hospitals or neighboring general hospitals where the cases originated.

The study was approved by relevant ethical review committees and participants signed written informed consent according to local regulations.

The questionnaire recorded demographic characteristics (age, sex, area of birth and residence history, religion), education (ever attendance to school, number of years of school attendance and age when schooling stopped), tobacco use and drinking habits, dietary habits, marital status, histories of various diseases and family history of cancer. The questionnaire also contained detailed information on smoking habits, including changes over time (number and kind of cigarettes) and smoking of other tobacco products (pipes and cigars). A smoker was defined as a subject reporting to have smoked daily for at least one year. The occupational history included job title, duration of the subject’s job, and for each job, the subject must have held the position for at least one year, detailed description of performed tasks, use of chemicals, branch of industry, company name and starting and ending years.

Information on the highest attained educations was reported by study subjects and harmonized across the cohorts. Industrial hygienists and other experts estimated the exposure to occupational agents based on the detailed occupational history collected via questionnaires. Employment in a given occupation or industry was defined as holding a job for at least one year in that occupation or industry. The cumulative duration of employment in a given occupation or industry, in years, was calculated by summing all jobs in that occupation or industry.

Details have been reported elsewhere [54]. We considered the mediating role of ever occupational exposure to (i) diesel exhaust, (ii) crystalline silica and (iii) welding fumes on the association between education (classified as low/medium vs. high) and lung cancer risk. Also, we calculated the mediating role of any occupational exposure among these three. Results on the three exposures and risks of lung cancer, as well as on the role of education as a risk factor, are reported in Table 3.

First, we estimated the odds ratios (OR) of lung cancer and the corresponding 95% confidence intervals (CI) through multivariable logistic regression models adjusted by country, sex, age, smoking status (never vs. ever tobacco smokers) and education.

For the mediation analysis, we decomposed the total effect of education into a natural direct effect, i.e., independent of the effect of occupation and a natural indirect effect, which reflects the mediation exerted by each carcinogen; then, we calculated the proportion of mediation (PM) as the ratio between the log of the natural indirect effect and that of the total effect [55,56]. The analysis was performed by using the commands logistic and paramed in STATA [57].

The study population included 2861 cases and 2936 controls. Table 3 summarizes the main characteristics of the study population, showing the distribution of the different occupational exposures between cases and controls. 

Table 4 illustrates the results of the mediation analysis. According to our analysis, 5 to 11% of the risk of lung cancer exerted by educational level was mediated by exposure to diesel exhaust, crystalline silica and welding fumes. Overall, for workers with any of these exposures, occupational carcinogens mediated about 14% of the association between education and lung cancer. Small differences were evidenced among the adjusted and the unadjusted models. 

The exposure investigated, namely diesel exhaust, crystalline silica and welding fumes, were selected as an example of occupational carcinogens of the lung. Indeed, they represent some of the main causes of occupational lung cancer [58]. The results of this analysis show that occupational exposures to diesel exhaust and welding fumes, and to a lesser extent, crystalline silica, mediate part of the risk exerted by educational level on lung cancer. That is, part of the reason why workers with low educational level have a higher likelihood of developing lung cancer depends on their occupational exposure to carcinogens. 

This analysis presents new data on the relationship between education and lung cancer, adding original insights on the role of occupational risk factors as a mediator of education’s effect. The case−control design and the lack of quantitative information on the exposures (which were expressed as dichotomous variables) represent limitations of this analysis. 

Further studies focused on the investigation of the mediating role of occupation in the association between education and occupational-related cancers would be of interest. 

## 4. Conclusions

Cancer disparities partially originate from occupational factors, including type of job, job position, working tasks, working schedule, salary and working status. Cancer screening participation varies across working categories, reflecting different access to health services. A proportion of the cancer inequalities rising from a difference in the level of education and from race/ethnicity can be explained through the same occupational-related factors, for example, because of differences in exposure to carcinogens at the workplace. 

We estimated that between 13.4% and 14.8% of the association between education and lung cancer is mediated by occupational exposure to diesel exhaust, crystalline silica or welding fumes. This implies that those subjects which are exposed to these carcinogens are predominantly from lower educational levels. 

Since some working categories are well known to be at a higher risk of cancer, their difficulty to access cancer screening and care should be better addressed, for instance, by implementing health policies at the workplace. This approach is consistent with the perspective of Total Worker Health [40], which does not limit the targeting of workers to those highly exposed to a particular carcinogen in the workplace, but rather addresses the entire working population, accounting for characteristics which may lead to poor health, including cancer. The two approaches are not oppositional, rather they are complementary: the workplace may become a setting to identify those workers with a high-risk profile (e.g., based on sociodemographic, lifestyle, family-related and clinical factors) for a particular disease, irrespective of the occupational nature of the disease itself.

Thus, the occupational physician should be aware of the causes and the consequences of occupational-related discrepancies in cancer health and control, in order to manage them [42]. An approach based on economic reward [30] for participating in cancer screening may be feasible in some subgroups of the working population, e.g., based on health insurance availability and quality, salary or exposure to occupational carcinogens. The use of specific indices and matrices to quantify the socioeconomic disparities may help to achieve this aim [59]. 

While occupational cancer hazards have been mainly studied in Whites, the evidence for other racial and ethnic groups is weak. Also, illiteracy should represent an alarm flag to consider when monitoring working populations at risk for cancer. 

The investigation of cancer discrepancies based on occupation-related factors would benefit from prospective studies including minority workers and should be designed to collect occupational data through validated instruments. 

Further studies are needed to explore the mediating role of occupation on shaping cancer disparities. For example, we plan to expand the preliminary analysis presented here regarding occupational carcinogens as mediators of the association between education and lung cancer risk. The introduction of cancer screening programs at the occupational level may represent a future direction for cancer preventive strategies.

## Figures and Tables

**Table 1:** Effect of different disparities factors into participation in cancer screening, based on selected studies.

Study	Country	Disparity Factors	Population	Findings			
Rajaguru et al., 2022 [11]	Korea	Education Employment Insurance Income	20,347, both sexes, aged 40 and older targeted for cancer screening; Korea National Health and Nutrition Examination Survey (KNHANES)	Use of cancer screening			
				University or over vs. elementary: 1.25, 1.02–1.47			
				Occupation vs. no occupation: 1.41, 1.15–1.73			
				Private vs. no private health insurance: 2.73, 1.50–4.94			
				Income Q4: 4.07, 1.63–10.13 (reference: Q1)			
Leinonen et al., 2017 [12]	Norway	Education Occupation Employment Income	Norwegian women targeted for cervical cancer screening	Percentages of non-adherence:			
				42% from primary school, 30% from university;			
				41% manual, trades, military occupation, 28% managerial occupation;			
				43% unemployed, 30% employed;			
				45% lowest income, 29% highest income			
Broberg G et al., 2018 [13]	Sweden	Income Education Employment	Women aged 30–60 targeted for cervical cancer screening. 314,302 cases (no smear for 6–8 years); 266,706 controls (smear within 90 days)	Predictors of non-adherence:			
				Disposable family income (<24.222 € vs. >50.111 €): 2.06, 2.01–2.11;			
				Low education: (≤9 years vs. ≥12 years) 1.77, 1.73–1.81;			
				Unemployment: 2.15, 2.11–2.19			
Shim HY et al., 2019 [14]	Korea	Occupation Working hours Shifts	5626, both sexes, aged 40 and over targeted for gastric cancer screening	Prediction of participation to screening:			
				Manual workers: 0.74, 0.55–0.99			
				Sales/service workers: 0.62, 0.47–0.81			
				Machine operators: 0.67, 0.50–0.91			
				vs office workers/clerk;			

				Part-time workers: 0.81, 0.67–0.99 vs. full-time workers;			

				≥60 working hours: 0.93, 0.78–1.11 vs. ≤40 h;			
				Shift workers: 0.87, 0.73–1.04 vs. day workers (adjusted for age, gender, smoking and alcohol)			
Shete S et al., 2021 [15]	USA (pooled analysis from 11 population-based surveys)	Insurance Education	2897 women aged 50–75 targeted for colorectal and breast cancer screening	Difference in cancer screening participation among US women			

				No difference by income in CCR and BC screening			

				CCR participation 82% in urban vs. 78% in rural residents, no difference in breast			

				CCR screening participation:			
				Private or employee-based health insurance: 1.99, 1.30–3.06 vs. no insurance			
				Medicare: 2.34, 1.43–3.84 vs. no insurance			
				Medicaid: 2.00, 1.15–3.49 vs. no insurance			
				Education ≥ college: 1.30, 0.99–1.71			
				vs. ≤high school			
				Post-high school trainings: 1.15, 0.88–1.51 vs. ≤high school			

				BC screening participation:			
				Private or employee-based health insurance: 3.80, 2.45–5.88 vs. no insurance			
				Medicare: 2.84, 1.81–4.47 vs. no insurance			
				Medicaid: 2.58, 1.47–4.52 vs. no insurance			
				Education ≥ college: 1.19, 0.90–1.58 vs. ≤ high school			
				Post-high school trainings: 1.17, 0.90–1.52 vs. ≤ high school			
Fedewa et al., 2017 [16]	USA	Occupational characteristics (occupation, industry type and employer size)	National Health Interview Surveys (NHIS) among eligible US workers (CC women 21–65 years; *n* = 20,997), (BC women ≥ 40 years; *n* = 14,258) and (CRC men and women ≥ 50 years; *n* = 17,333)	Higher rates of colonoscopy in larger employers (500+ workers),			
				lower rates in smaller size employers (1–24 workers)			
				Insured employees % positively related to employer size			

				Participation to CC screening:			
				<50% in construction, food service,			
				production/transport, healthcare/personal support workers;			
				66% in scientists and educators			

				Higher % of uninsured in construction			
				and production/transport workers (also with lower adherence to cancer screening).			

				(Ref =Healthcare practitioners)			
				Food service	0.94	0.9	0.98
				Construction	0.91	0.87	0.95
				Sales	0.94	0.9	0.97
				Office support	0.97	0.95	1
				Production	0.95	0.91	0.98
Carney et al., 2012 [17]	USA	Insurance	Oregon Rural Practice-based Research Network (ORPRN)	Up-to-date BC screening status clinical breast examination:			
				Medicare/Medicare plus private: 1.63, 1.04–2.56			
				Medicaid/Medicaid plus private: 0.98, 0.41–2.31			
				Uninsured: 0.76, 0.39–1.48			

				Mammography:			
				Medicare/Medicare plus private: 0.73 (0.53–1.02)			
				Medicaid/Medicaid plus private: 0.67 (0.41–1.09)			
				Uninsured:0.44 (0.24–0.79)			

				CC screening			
				Medicare/Medicare plus private: 0.62 (0.25–1.55)			
				Medicaid/Medicaid plus private: 0.79 (0.24–2.58)			
				Uninsured: 0.48 (0.19–1.24)			

				CCR screening			
				Medicare/Medicare plus private: 0.77 (0.53–1.10)			
				Medicaid/Medicaid plus private: 0.60 (0.34–1.05)			
				Uninsured:0.43 (0.19–1.00)			
Ishii et al., 2021 [18]	Japan	Education Employment status	2016 Comprehensive Survey of Living Conditions of People on Health and Welfare, a national cross-sectional survey conducted by the Japanese Ministry of Health, Labor and Welfare. Japanese women targeted for CC, BC and CRC screening 115,254 aged 40–69	Participation to CC, BC and CRC screening:			
				*Educational attainment*	CC	BC	CRC
				University	54.6	56.1	46.3
				College/vocational school	48.3	48.4	41.7
				High school	42.4	43.9	39.6
				Junior high school	28.4	29.5	30.5
				*Employment status*			
				Permanent worker	58.7	59.9	53.9
				Contracted worker	53.6	55.1	50.2
				Dispatched worker	46.3	46.9	34.1
				Part-time worker	44.3	44.9	37.1
				Self-employed/other	42.6	43.6	37.6
				Homemaker	39.7	41.4	36.5
				Not working	29.5	32	31.6
Tapera et al., 2019 [19]	Zimbabwe	Education Occupation Personal income Household income Wealth quintile	143 women aged 25 and older targeted for cervical cancer screening	Education			
				Primary	0.22	to 895	
				Secondary	2.14	0.23 to 19.82	
				Higher	–	–	
				None			
				Occupation			
				Unemployed	0.1	0.01 to 1.60	
				Professional	0.84	0.05 to 13.11	
				Self-employed	Ref	–	
				Other	0.67	0.02 to 22.98	
Amin et al., 2020 [20]	Iran	Education Employment status Insurance		*Education*			
				Illiterate	1		
				1–6 years	1.76	1.531	2.029
				6–12 years	2.47	2.088	2.932
				>12 years	2.24	1.803	2.786
				*Employment status*			
				Unemployed	1		
				Employed	0.83	0.714	0.986
				Retired	1.07	0.713	1.622
				Student	0.92	0.423	2.019
				*Insurance*			
				No	1		
				Yes	1.5	1.245	1.808

BC = breast cancer; CC = cervical cancer; CRC = colorectal cancer. Ref = reference category.

**Table 2:** Selected studies reporting information on education and race/ethnicity as a reason for occupational disparities.

Ref.	Outcome	Type of Cancer	Population	Industry/Type of Exposure	Data	Finding
Michaels D, 1983 [43]	Mortality	Lung	Black	Steel workers	89% of Blacks working in coke plants were employed in ovens vs. 31% of Whites	Three times higher lung cancer mortality in Blacks than in Whites employed in the same coke plants.
	Incidence	Stomach, lung, blood, bladder, lymphatic and prostate	Black	Rubber industry	27% of Blacks working on mixing and compounding vs. 3% of Whites	Elevated risk of stomach, lung, blood, bladder, lymphatic and prostate cancer in mixing and compounding workers. Two times higher rates of lung and prostate cancer in Blacks than in Whites working in the same area.
	Mortality			Shipyards	38% of the shipyard workforce at the end of World War II were Black	High mortality for asbestos-related cancers.
	Incidence	Lung	Black	Foundry	More than 25% of foundry workers were Black at the end of World War II	Black foundry workers are at a greater risk than the industry’s White workers.
Juon HS et al., 2021 [44]	Cancer incidence; prevalence of exposure to carcinogens of the lung	Lung	Black	NA	Black vs. White Lung cancer incidence: 4.3% vs. 3.9%; OR=1.24, 1.01–1.53 (adjusted for smoking and pack-years) Overall exposure prevalence: 32% vs. 28% - Silica: 10% vs. 6.3% - Asbestos: 7% vs. 4.5% - Foundry/steel mining 7.7% vs. 4.1% - Painting 7.8% vs. 5% Similar stage at diagnosis.	Blacks seem to need a particular protection and need to be addressed with educational programs at the workplace.
Boyle et al., 2015 [45]	Occupational exposure	NA	Ethnic minorities in Australia	NA	Marked difference in the exposure to the overall carcinogens (*p* < 0.001), particularly high among Arabic people not speaking English. Higher solar radiation and diesel exhaust in Arabic; higher environmental tobacco smoke in Chinese; higher polycyclic aromatic hydrocarbons in the Vietnamese population.	Targeted and informed occupational health and safety measures to be implemented based on the different prevalence of exposure to occupational carcinogens by ethnic groups.
Carey RN et al., 2021 [46]	Occupational exposure	NA	Ethnic minorities in Australia	Exposure to benzene, diesel engine exhaust, environmental tobacco smoke, ionizing radiation, lead, polycyclic aromatic hydrocarbons other than vehicle exhausts, graveyard shiftwork, silica, solar ultraviolet radiation and wood dust	79% of Māori/Pasifika workers vs. 67% of New Zealand Caucasian workers were exposed to at least one occupational carcinogen.	Ethnic disparities in occupational exposure to carcinogens after migration to Australia. Māori/Pasifika workers were more likely to report exposure to carcinogens, in particular environmental tobacco smoke.
Gosselin A et al., 2020 [47]	Occupational exposure	NA	Australian immigrants born in New Zealand, India and Philippines	Exposure to solar and artificial ultraviolet radiation, diesel engine exhaust, environmental tobacco smoke, benzene, lead, silica, wood dust, other polycyclic aromatic hydrocarbons and shift work	Risk of exposure to at least one occupational carcinogen in New Zealand workers compared to Indian: 1.61, 1.12–2.32. Diesel exhaust exposure in New Zealand workers compared to Indian: 2.61, 1.60–4.25.	The prevalence of exposure to workplace hazards varied by both social position and occupational characteristics. Disparities in exposure to some workplace hazards occurred among this population as a result of their social position and irrespective of the type of job they undertook. The most vulnerable groups for exposure to carcinogens were young workers who worked long hours in smaller companies, particularly if they were born in New Zealand. Examining occupational characteristics alone may hide discrepancies related to exposure to carcinogens among workers. Subgroups of workers may have a particularly high exposure to carcinogens.
Pokhrel A et al., 2010 [48]	Cancer survival	NA	Finland	NA	In 1996–2005, 4–7% of the deaths in Finnish cancer patients could have been avoided in the 5 years after diagnosis, if all the patients had the highest educational background.	High survival rates in highly educated and highly health-conscious people; low survival rates in those with low education; less favorable distribution of tumor stages in the lower education category. In 1996–2005, 8–11% of first 5-year cancer deaths would have been avoided if all the patients had the same cancer and the mortality for other causes had been the same as that in the highest educational category.
Menvielle G et al., 2010 [49]	Occupational exposure	Lung	Men, EPIC cohort (Denmark, the United Kingdom, Germany, Italy, Spain and Greece)	Exposure to asbestos, heavy metals and polycyclic aromatic hydrocarbons	After adjustment (smoke and fruits/vegetables), occupation explained 14% of the excess risk. Relative incidence of inequalities: 1.75 (1.27–2.41) after adjusting for tobacco smoking, fruit and vegetable consumption.	A common hypothesis is that a higher exposure to risk factors explains the higher incidence of lung cancer in low socioeconomic groups. The risk factors are seen as intermediate variables or mediators between education and the onset of lung cancer. Birth cohort analyses suggest an effect of occupational exposures among older men but not younger men on educational inequalities.

**Table 3:** Selected characteristics of the study population.

Characteristics	Lung Cancer Cases	Controls	OR, 95% CI
Smoking status			
-Never	274 (9.6%)	1038 (35.4%)	Ref
-Former	1310 (45.8%)	995 (33.9%)	6.27, 5.27–7.47
-Current	1277 (44.6%)	900 (30.7%)	6.67, 5.59–7.96
Diesel exhaust *			
-No	2108 (73.7%)	2289 (78.0%)	Ref
-Yes	753 (26.3%)	647 (22.0%)	1.15, 1.01–1.33
Crystalline silica *			
-No	2694 (94.2%)	2827 (96.3%)	Ref
-Yes	167 (5.84%)	109 (3.71%)	1.75, 1.33–2.31
Welding fumes *			
-No	1783 (62.3%)	2013 (68.6%)	Ref
-Yes	1078 (37.7%)	923 (31.4%)	1.18, 1.04–1.35
Education †			
-Low	402 (14.1%)	582 (19.9%)	Ref
-Medium	2026 (70.9%)	2007 (68.5%)	1.34, 1.15–1.56
-High	427 (15.0%)	341 (11.6%)	1.74, 1.38–2.19

* ever exposure; † based on country-specific cut-points (see [54] for details).

**Table 4:** Analysis of the mediating effect of selected occupational carcinogens on the association between education and lung cancer.

	OR and 95% CI of Lung Cancer							
	Diesel Exhaust		Crystalline Silica		Welding Fumes		Any	
	aOR (95% CI)	OR (95% CI)	aOR (95% CI)	OR (95% CI)	aOR (95% CI)	OR (95% CI)	aOR	OR
NDE	1.26 (1.04–1.53)	1.29 (1.11–1.51)	1.27 (1.05–1.53)	1.33 (1.14–1.54)	1.26 (1.04–1.52)	1.29 (1.10–1.50)	1.42 (1.20–1.67)	1.28 (1.10–1.49)
NIE	1.02 (0.98–1.06)	1.04 (1.01–1.06)	1.01 (1.00–1.02)	1.01 (1.00–1.02)	1.03 (0.97–1.09)	1.04 (1.02–1.06)	1.06 (1.00–1.12)	1.04 (1.02–1.07)
TE	1.29 (1.06–1.56)	1.34 (1.15–1.56)	1.28 (1.06–1.55)	1.34 (1.15–1.56)	1.29 (1.06–1.57)	1.34 (1.25–1.56)	1.50 (1.26–1.78)	1.34 (1.15–1.56)
PM	7.8%	12.5%	4.9%	3.4%	10.8%	13.3%	13.4%	14.8%

OR, odds ratio. aOR = adjusted odds ratio; adjusted for country, sex, age and smoking status. CI, confidence interval. NDE, natural direct effect. NIE, natural indirect effect. TE, total effect. PM, proportion of mediation. Any = exposure to diesel/kerosene or silica or welding.
